# Time Pattern Locking Scheme for Secure Multimedia Contents in Human-Centric Device

**DOI:** 10.1155/2014/796515

**Published:** 2014-08-17

**Authors:** Hyun-Woo Kim, Jun-Ho Kim, Jong Hyuk Park, Young-Sik Jeong

**Affiliations:** ^1^Department of Multimedia Engineering, Dongguk University, Seoul 100-715, Republic of Korea; ^2^Department of Computer Science and Engineering, Seoul National University of Science and Technology, Seoul 139-743, Republic of Korea

## Abstract

Among the various smart multimedia devices, multimedia smartphones have become the most widespread due to their convenient portability and real-time information sharing, as well as various other built-in features. Accordingly, since personal and business activities can be carried out using multimedia smartphones without restrictions based on time and location, people have more leisure time and convenience than ever. However, problems such as loss, theft, and information leakage because of convenient portability have also increased proportionally. As a result, most multimedia smartphones are equipped with various built-in locking features. Pattern lock, personal identification numbers, and passwords are the most used locking features on current smartphones, but these are vulnerable to shoulder surfing and smudge attacks, allowing malicious users to bypass the security feature easily. In particular, the smudge attack technique is a convenient way to unlock multimedia smartphones after they have been stolen. In this paper, we propose the secure locking screen using time pattern (SLSTP) focusing on improved security and convenience for users to support human-centric multimedia device completely. The SLSTP can provide a simple interface to users and reduce the risk factors pertaining to security leakage to malicious third parties.

## 1. Introduction

The smart multimedia devices that have been developed through active and continuous research on IT have penetrated deeply into their users' daily living. In particular, multimedia smartphones provide users with basic functions such as an alarm, memos, contacts, and camera, as well as having various other features. In this way, they increase leisure time utilization and convenience [1–5]. In addition, it is also utilized for various purposes such as networking services (e.g., chatting, social networking service, blog, email, etc.) for real-time communication with other users, work processing, and multimedia services (e.g., video chatting, multimedia device control, or multimedia data sharing). For these reasons, the penetration rate of the multimedia smartphone has continuously increased. According to the statistics provided by Strategy Analytics (SA), an IT market research institution, the number of the multimedia smartphone users currently exceeds 1 billion. However, since the multimedia smartphones are taken everywhere by users always because of its convenient features, they are likely to be lost or stolen. As a result, most multimedia smartphones are now equipped with various built-in locking features [6–11]. These include a personal identification number (PIN), which uses a simple number combination; a password, which uses numbers and characters; pattern lock via dragging in the preferred direction; face recognition of the user; face and voice recognition of the user; and drag to hide the screen. Among these, pattern lock, PINs, and passwords are the most widely used locking features, but they are highly vulnerable to shoulder surfing and smudge attacks, and therefore a new type of locking system is required [12–14]. Therefore, the enhanced lock function is required to be processed multimedia content effectively due to increase in the popularity of smart devices.

In this paper, we propose a secure locking screen using time pattern (SLSTP) focusing on the security of the multimedia smartphone and the convenience of users for supporting human-centric multimedia device completely. The main motivation of this research is an effective locking mechanism by adding the logical pattern with enhanced security features in order to compensate for the fragility of the locking device of the existing smart devices. The SLSTP utilizes pressing time on the touch screen, as well as pressing counts. While providing convenience to users due to its simple control, the SLSTP makes it difficult for malicious third parties to infer the pattern easily because the pressing time is also included.

The remainder of this paper is organized as follows. In Section 2, basic built-in locking features of multimedia smartphones are discussed. In Section 3, a time pattern setup method and time pattern encryption method used in the SLSTP are explained. In Section 4, the design of the SLSTP is addressed, while the implementation of the SLSTP is discussed in Section 5.  Section 6 provides a comparison of the security strength of the SLSTP with that of the existing locking features. Finally, conclusions and directions for future research are given in Section 7.

## 2. Related Work

In this section, the pattern lock, face recognition, face and voice recognition, PIN, and password approaches, which are basic locking features embedded in multimedia smartphones, are briefly explained [15, 16].  Table 1 illustrates the basic built-in locking features in multimedia smartphones.

## 3. Scheme of the SLSTP

Most traditional locking functions are provided by means of a physical basis such as simple touch or drag. Such methods are vulnerable to smudge or shoulder surfing attacks, which represent attack techniques for smartphones. In addition, password or PIN methods are highly vulnerable to brute force attack. To overcome such physical vulnerability, the SLSTP applied time, which is a logical unit. The time pattern setup and encryption of the SLSTP are explained in the following section.

### 3.1. Time Pattern Schema

**Figure 1:**
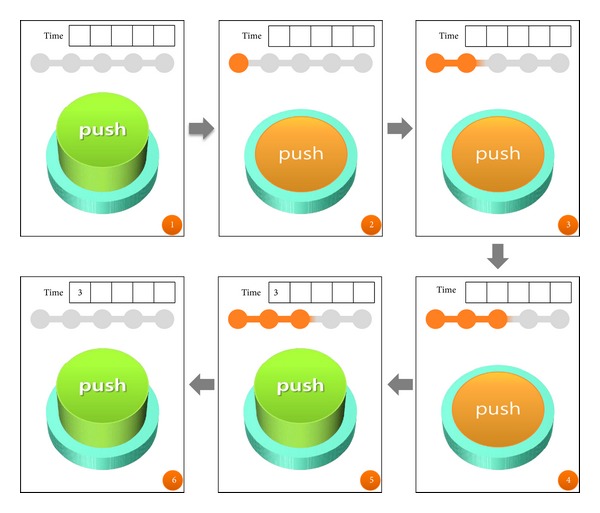
The time pattern input procedure of SLSTP.

The SLSTP proposed in this study sets the locking feature using the pressing time on the screen of a multimedia smartphone, along with the number of presses.  Figure 1 shows the procedure for inputting a time pattern in the SLSTP as follows: ① in Figure 1 represents the standby status to receive a time pattern from a user. ② shows one second elapsed time after a user touches the button. Here, the bar at the upper end shows a pressing time until now to a user. ③ shows two-second elapsed time after the touch button is pressed and held down, while ④ shows three-second elapsed time. ⑤ shows the releases of the touch, resulting in the first input of a time pattern. ⑥ shows another standby status to receive another input from a user after three seconds recognized from ⑤. In this manner, a time pattern is received from the user.

To unlock the screen, any place on the multimedia smartphone can be touched to input the pattern. Since the SLSTP uses a simple pressing time and number, it is convenient for the user.

The pressing time is measured starting from the time a user touches and holds the space on the screen until the touch is released. Since the precision of the time measured can be refined according to orders of magnitude of different times, it is highly difficult to infer a set time pattern. In addition, the SLSTP allows a number of inputs so that it provides improved security against smudge attack, which is the typical touch screen-based attack method.

### 3.2. Encryption of Time Pattern

**Figure 2:**
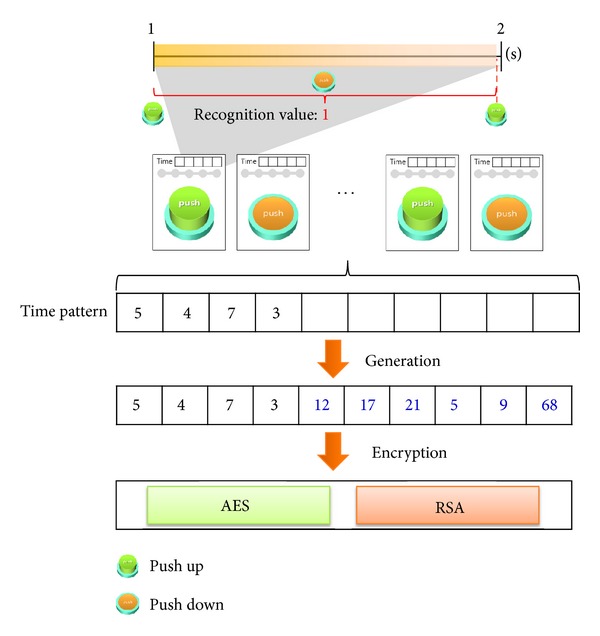
Time pattern recognition and encryption of SLSTP.

The SLSTP analyzes a time pattern entered by a user and estimates whether this pattern can be inferred by a malicious attacker. First, it compares the input time pattern with user information, thereby determining whether or not they are the same. If they are the same, the system gives a new input request to a user. If a time pattern is inputted that is not related to the user information, encryption is performed. Two encryption methods are used: the AES (Advanced Encryption Standard) algorithm and the RSA (Rivest-Shamir-Adleman) algorithm. If a key count of the input time pattern is less than 10, it is modified to become at least 10 through the random number generation. In addition, the SLSTP does not limit an input length of time pattern from a user, so that it can utilize up to all of the available Read Access Memory. If an inputted time pattern is long, the system partially stores the encrypted pattern.  Figure 2 shows the time pattern recognition and encryption for the SLSTP. The recognition value is displayed in the upper side of Figure 2; it shows when the absolute value of time pattern was set to one second. If a push button is released within one to two seconds, the recognition value is one second. An absolute value during this operation can be modified to 0.5 seconds or two seconds via the user's setup. In addition, users may have difficulty recognizing a short time, such as 0.5 seconds, so vibration can be provided to help users recognize the time progress.

## 4. Design of SLSTP

**Figure 3:**
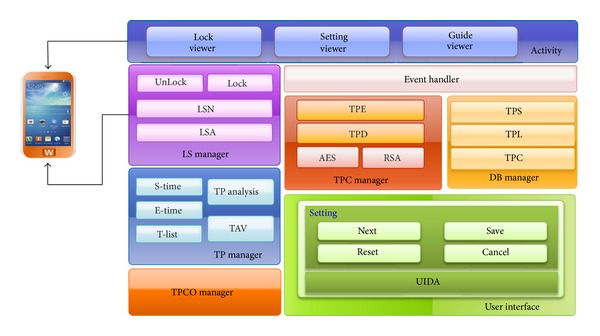
Overall architecture of SLSTP module structure for locking multimedia contents.

The secure locking system using time pattern (SLSTP) proposed in this study consists of a user interface to set up a time pattern and a locking release by a user, a TP Manager to analyze the time patterns, a TPC Manager for time pattern encryption, a DB Manager to store the encrypted time patterns, an LS Manager to manage screen locking and unlocking of the SLSTP in smartphones, a TPCO Manager to manage locking the screen release in case the SLSTP is activated, an Event Handler for organic communication between Managers and Activity, and Activity to show the locking screen for users.  Figure 3 shows the overall architecture of the SLSTP.

*User interface* consists of Setting, which is used to setup a time pattern by a user, and UIDA (user input detect area), which detects the user's input. In Setting, the following buttons are available: Next, Save, Reset, and Cancel. The Next button plays a role in inputting a time pattern by a user for locking the setup and changing the input state to the reentry request state. Once the same time pattern is inputted into the reentry state, the time pattern is applied to the locking screen through the Save button. The Reset button plays a role in returning to the initial state if a user sets up a time pattern incorrectly. The Cancel button plays a role in terminating the setup when a user does not want to setup the SLSTP for a locking screen. UIDA plays a role in inputting a time pattern by a user when the screen locking function of the SLSTP is activated.

*TP Manager *(time pattern manager) consists of S-Time (start time), which reads the first time button that is pressed once the input is detected via the user interface and E-Time (end time), which reads the button release time when the pushed button is released. A pressing time is calculated by the difference between S-Time and E-Time, and that value is stored in T-List (time list). TP Analysis (time pattern analysis) in TP Manager analyzes the measured values of the pressing times through the TAV (time absolute value). The TAV represents an allowable time for determining an absolute value of the time difference.

*TPC Manager *(time pattern cipher manager) consists of TPE (time pattern encryption), which plays a role in encrypting the time patterns received from the TP Manager and TPD (time pattern decryption), which plays a role in decrypting the encrypted time patterns. Two encryption methods are used: AES (advanced encryption standard) and RSA (Rivest-Shamir-Adleman).

*DB Manager* plays a role in storing, loading, and comparing the time patterns encrypted in the TPC Manager in smartphones. The DB Manager consists of TPS (time pattern save), which saves a time pattern, TPL (time pattern load), which loads a time pattern, and TPC (time pattern compare), which compares an input time pattern and the saved time patterns to unlock the locked screen. TPC organically communicates with the TPC Manager to compare an input time pattern with the saved encrypted time patterns.

*LS Manager *(lock screen manager) is responsible for locking and unlocking the screen in smartphones. The LS Manager consists of LSA (lock state analysis), which analyzes a locking state, and LSN (lock state notification), which applies a locking or unlocking state to smartphones. Through the LSN, Lock, which performs screen locking, and UnLock, which releases screen unlocking, are called each function.

*TPCO Manager *(time pattern count operation manager) is responsible for counting the number of incorrect time patterns that are inputted by a user when the SLSTP is activated. If a user enters an incorrect time pattern five times in a row, the TPCO Manager changes the screen into a locking state for 30 seconds. After 30 seconds has elapsed, it is reinitialized to count again. The count number can be adjusted based on the user's preference.

*Event Handler* plays a role as a broker to share information organically between Activity and the other managers, such as the TP Manager, the LS Manager, and the TPCO Manager. Through the Event Handler, all the situations performed in Activity can be reflected.

*Activity* consists of the Guide Viewer, which shows a novice SLSTP user how to use the system, the Setting Viewer, which sets a count for the number of allowable input times in a row and a time pattern for the SLSTP, and the Lock Viewer, which shows the SLSTP activation for users.

## 5. Implementation of SLSTP 

**Figure 4:**
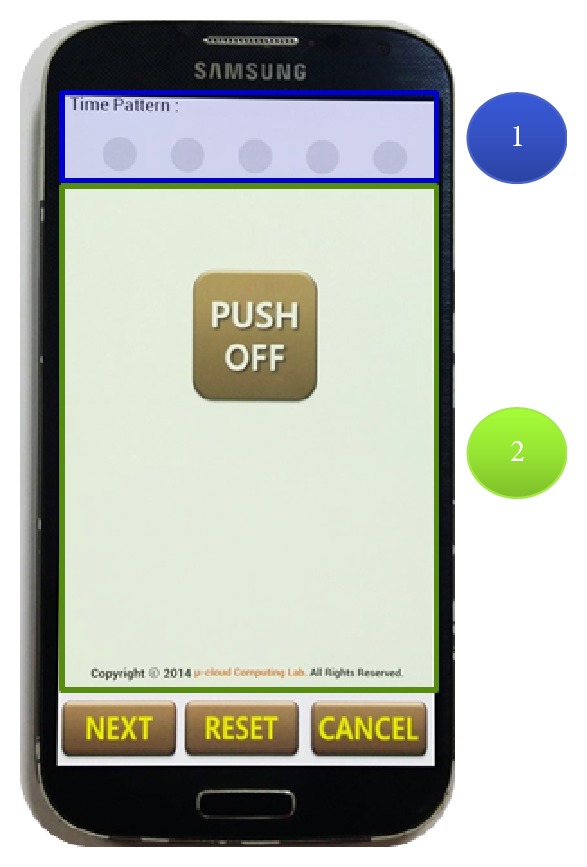
Initial execution screen of SLSTP.

The initial screen of the SLSTP proposed in this paper is shown in Figure 4. ① of Figure 4 shows a button to input the time pattern, while ② shows the status bar exhibiting the user's pressing time. Through the Next button, a reinput screen comes up to confirm whether the set input time pattern is correct. Cancel is used when a user does not want the SLSTP locking feature to be activated.

**Figure 5:**
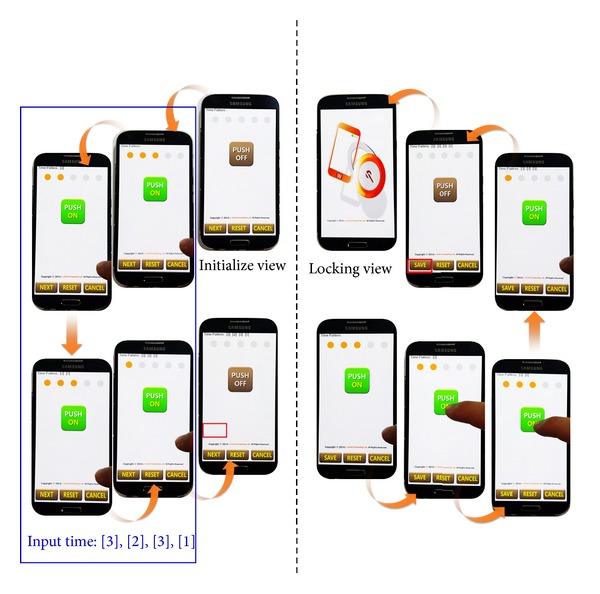
Setup process locking mechanism of SLSTP.

Figure 5 shows setup process locking mechanism of SLSTP. The left screen in Figure 5 shows an input screen for a time pattern to be set by the user. For example, four inputs of 3 sec, 2 sec, 3 sec, and 1 sec are entered followed by pressing down the Next button to change to the next screen. The right screen in Figure 5 shows an activated screen after the Next button is pressed, in which previously entered same time pattern is entered. This will determine whether the time pattern that the user entered is correct. If the time pattern is not the same as the previous one, reinput is required. If three attempts have been made without success, then the process is reset and the input starts again. If all inputs are complete, the Save button at the bottom is pressed to save the time pattern. The entered time pattern is encrypted internally. As an encryption method, the data encryption standard (DES), advanced encryption standard (AES), or Hash method can be selected according to the password strength.

**Figure 6:**
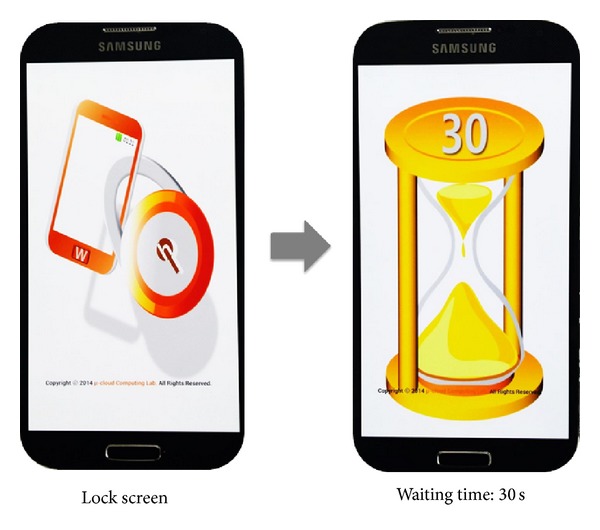
Waiting visualization for nonauthorizing access of SLSTP.

Figure 6 shows waiting visualization for nonauthorizing access of SLSTP. The left screen in Figure 6 shows the activated status of the locking screen of SLSTP, while the right screen shows an activated status when a user enters an incorrect time pattern five times consecutively. The upper bar indicates the standby time for the next attempt.

## 6. Performance Evaluation

In this section, the SLSTP proposed in this paper is compared with the other popular locking systems, such as pattern lock, password, and PIN.

The number of locking patterns using pattern lock is limited because it sets a password using the static 3-by-3 point approach. In addition, pattern lock, password, and PIN require a minimum of four password keys to ensure basic security strength. Password and PIN are both limited to setting password keys of no more than 16 digits or characters.  Table 2 shows the comparison of the SLSTP with other popular locking systems in terms of the pattern lock, password, and PIN limitations. For the password, uppercased and lowercased letters and numbers (except for special characters) were considered. As shown in Table 2, the number of locking patterns that can be setup increases as the number of password keys increases. This means the number of patterns that can be set for the number of password input keys. However, pattern lock can only employ a key length of up to nine digits or characters, so it is not comparable to other password key options that can use more than 10 digits or characters. In addition, as the key length increases, password shows higher security strength in comparison to PIN and pattern lock. Moreover, as the key length increases it becomes more difficult for a user to remember his/her password, resulting in inconvenient password entry. The SLSTP proposed in this study employs time as a logical unit so that it can provide an unlimited number of locking patterns for any key length. The SLSTP has high security strength due to its differentiation from other locking functions because it measures time despite of the fact that it only uses one button push.

**Figure 7:**
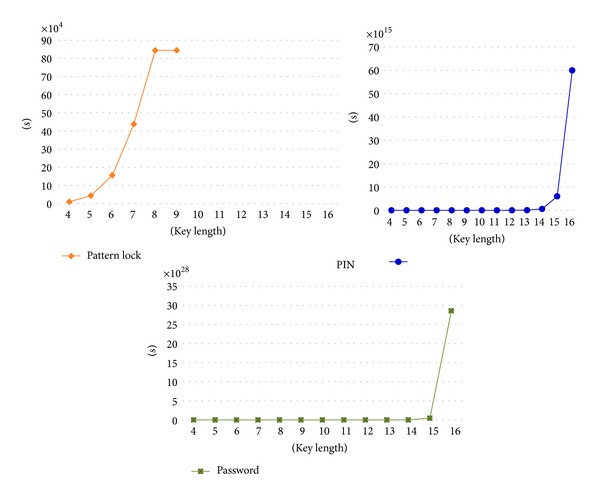
Timing for all possibility patterns depending on pattern length.

Figure 7 shows a result that applied 30-second delay due to five incorrect inputs of a password pattern using pattern lock, password, and PIN. It means the worst (maximum) amount of time when released pattern based on the number of password input keys. Since it is difficult for the SLSTP to estimate the time pattern of a single pressing, as shown in Table 2, pattern lock, password, and PIN were measured for this experiment. As shown in Figure 7, the time taken to unlock the screen increases according to the set input key. However, the patterns using such methods are vulnerable to a brute force attack since the time taken to unlock the screen is limited by the maximum setup input key length.

On the other hand, since the number of locking patterns using the SLSTP is unlimited, the SLSTP can provide robust security strength to users. In addition, since it provides a simple interface for time measurement, it can also provide ease of use for users. While SLSTP can provide high security strength due to the use of time, it may take longer for the screen to be unlocked. Therefore, it is important for the user to setup the right time difference.

## 7. Conclusion

Although multimedia smartphones have become very popular among the general public thanks to their simple portability and various convenient features, the risk of important data loss due to phone loss or theft by a malicious third party has also increased. For these reasons, users of multimedia smartphones employ the built-in locking features in the multimedia smartphone. However, typical locking features have low security strength and are vulnerable to the shoulder surfing and smudge attacks, where passwords can be determined easily.

We proposed the SLSTP to provide convenience and improved security to users for supporting human-centric multimedia device completely. The SLSTP showed the strongest security against a smudge attack compared with the pattern lock, password, and PIN, which are the most frequently used security approaches in multimedia smartphones. Since the SLSTP uses logical data, the locking feature in the smart multimedia device can be strengthened, making it more difficult to conjecture the password pattern. Furthermore, the SLSTP provides enhanced usability because it is structured with a simple interface that involves simple pressing and releasing.

In the future, we will measure the user's pressing time more precisely to increase the security strength, which is proportional to time precision. Moreover, we will focus on a locking technique using a weak vibration that can only be felt by the user.

## Figures and Tables

**Table 1:** Basic types of built-in locking features in multimedia smartphones.

Locking system	Description
Pattern lock [15]	This locking feature is the most widely used by the general public. The pattern locking feature consists of a 3 × 3 grid with simple user interface. The user selects the starting point and drags the pattern. However, the number of patterns provided is limited. This feature is vulnerable to smudge attacks by malicious attackers to unlock the pattern.

PIN [16]	This locking feature is used by existing feature phones. In the conventional PIN, only four numbers could be entered. Now, a PIN may comprise a minimum of 4 up to 16 numbers. It has a weakness in that if a user sets up numbers that are related to the user (such as a birthday), it will be easier to unlock. Therefore, random numbers and longer PINs make the code stronger. However, the positions of the input numbers are always fixed on the screen so that it is vulnerable to smudge attacks. In addition, it is difficult to memorize the excessively long numbers that are needed for stronger security.

Password [16]	The password is the most widely used locking feature, not only for smartphones but also for logging in for email, home page, and SNS use. This locking feature can include a combination of various numbers, letters, and special characters. However, if the password chosen is the same as one which has been previously used, it may be exposed easily. In addition, password syndrome might occur if different passwords are used in situations where a login is required.

Face recognition [16]	This is a locking feature linked to the user's face; it employs the smartphone's built-in camera. To set up the feature, the user's face is captured in the face recognition boundary to be used for the locking system. To unlock the system, a facial expression similar to the setup face has to be recognized. This locking feature is vulnerable if malicious users provide a similar facial expression or have photos of the legitimate user.

Face and voice recognition [16]	In order to overcome the vulnerability of face recognition, face with voice is used as a locking feature. For its setup for locking, it follows the face recognition method along with a voice recording of the repeated pronunciation of set words. However, problems similar to those related to face recognition exist, where a similar face or photo can be used for facial recognition. Voice recognition can also be unlocked using voice modulation or a recorded voice. Thus, the usage frequency of this method is very low. Furthermore, if face or voice recognition does not work on the first attempt, the user has to attempt to unlock the device multiple times, which is inconvenient.

**Table 2:** The number of locking patterns according to the key length.

Key length	PIN	Pattern	Password	SLSTP
4	10000	1624	14776336	*∞*
5	100000	7152	916132832	*∞*
6	1000000	26016	56800235584	*∞*
7	10000000	72912	3521614606208	*∞*
8	100000000	140704	218340105584896	*∞*
9	1000000000	140704	13537086546263600	*∞*
10	10000000000	—	839299365868340000	*∞*
11	100000000000	—	52036560683837100000	*∞*
12	1000000000000	—	3226266762397900000000	*∞*
13	10000000000000	—	200028539268670000000000	*∞*
14	100000000000000	—	12401769434657500000000000	*∞*
15	1000000000000000	—	768909704948767000000000000	*∞*
16	10000000000000000	—	47672401706823500000000000000	*∞*
